# Improved Control of Tuberculosis and Activation of Macrophages in Mice Lacking Protein Kinase R

**DOI:** 10.1371/journal.pone.0030512

**Published:** 2012-02-16

**Authors:** Kangyun Wu, Jovanka Koo, Xiuju Jiang, Ran Chen, Stanley N. Cohen, Carl Nathan

**Affiliations:** 1 Department of Microbiology and Immunology, Weill Cornell Medical College, New York, New York, United States of America; 2 Department of Genetics, Stanford University School of Medicine, Palo Alto, California, United States of America; University of Maryland, United States of America

## Abstract

Host factors that microbial pathogens exploit for their propagation are potential targets for therapeuic countermeasures. No host enzyme has been identified whose genetic absence benefits the intact mammalian host in vivo during infection with *Mycobacterium tuberculosis* (Mtb), the leading cause of death from bacterial infection. Here, we report that the dsRNA-dependent protein kinase (PKR) is such an enzyme. PKR-deficient mice contained fewer viable Mtb and showed less pulmonary pathology than wild type mice. We identified two potential mechanisms for the protective effect of PKR deficiency: increased apoptosis of macrophages in response to Mtb and enhanced activation of macrophages in response to IFN-gamma. The restraining effect of PKR on macrophage activation was explained by its mediation of a previously unrecognized ability of IFN-gamma to induce low levels of the macrophage deactivating factor interleukin 10 (IL10). These observations suggest that PKR inhibitors may prove useful as an adjunctive treatment for tuberculosis.

## Introduction

In an era when the spread of antibiotic resistance has outpaced the introduction of new anti-infectives, attention has turned to the possibility of directing adjunctive anti-infective therapy against temporarily dispensable targets in the host [1]. If a drug does not act on the pathogen, the pathogen cannot become resistant based on the usual mechanisms: impaired drug uptake or retention, reduced drug activation, increased drug inactivation, or the mutation, over-expression or bypass of the target. This notion has lent increased interest to studying the biology of host-pathogen relationships by identifying cellular (host) genes exploited by pathogens (CGEPs) [2], [3].

The first CGEPs for a mycobacterium were identified when an RNAi screen confirmed the importance of phagocytic recognition and uptake machinery for *M. fortuitum* infection of a cell line from drosophila [4]. A CGEP for Mtb, the leading single cause of death from bacterial infection, emerged with the demonstration that protein kinase B (PKB; Akt) was required for optimal growth of Mtb in primary human macrophages in vitro [5]. However, the importance of this pathway in tuberculosis has apparently not been tested in an animal model. More recently, RNAi screens against all known kinases and phosphatases in a mouse macrophage cell line [6] and against all genes in a human macrophage cell line [7] identified numerous candidate CGEPs for Mtb.

Classical macrophage activation protects the host from diverse facultative or obligate intracellular pathogens, including Mtb. The major inducer of classical macrophage activation is IFN-gamma [8], [9]. In activated macrophages, IFN-gamma co-induces transcription of a major anti-mycobacterial effector enzyme, the Ca^2+^-independent isoform of nitric oxide synthase (iNOS) [10], [11]. However, certain cytokines can prevent, suppress or reverse macrophage activation. In order of their discovery, macrophage deactivation factors include a glycoprotein secreted by tumor cells [12], TGF-beta [13] and IL10 [14], [15]. IL10 is produced not only by T cells but also by macrophages themselves. IL10 antagonizes not only macrophage responses to IFN-gamma but also the production of IFNγ by T cells [16].

The pathogenesis of tuberculosis depends on the host's immune response in two competing ways. The Th1 immune response and ensuing macrophage activation restrain Mtb replication well enough that immunocompetent people with a skin test indicative of persistent infection face only a 5–10% chance of developing clinically apparent tuberculosis. Yet survival of Mtb as a species requires that immunopathology progress far enough in some of those infected for host enzymes to liquefy lung tissue and generate an infectious aerosol [17]. Once host-mediated immunopathology is advanced enough to be recognized as active tuberculosis, it will kill about half of those affected unless they are treated. Thus, to survive as a species, humans must not only be able to activate their macrophages in response to this widespread pathogen but also deploy counter-regulatory mechanisms to restrain the immunopathologic response [18].

A screen for macrophage clones whose expression of certain genes was regulated by an expressed sequence tag library [19] led us to explore dsRNA-dependent protein kinase (PKR) as a candidate gene for affecting the cells' response to infection with Mtb (unpublished data). PKR is a widely expressed serine/threonine kinase whose expression is enhanced in response to type I IFN. Binding of dsRNA promotes PKR's homodimerization [20], [21], autophosphorylation and activation [22]. PKR-dependent phosphorylation of eukaryotic initiation factor 2-alpha impairs protein synthesis, contributing to IFN's antiviral actions [23]. However, PKR has many other activators, including LPS, IL1 and TNF-alpha [24], [25], and substrates, including insulin receptor substrate [26]. Despite PKR's potentially widespread actions, its genetic disruption appears to leave mice in good health. Moreover, PKR^−/−^ mice have displayed very limited phenotypes upon challenge with some viruses and no phenotype with others [27]. Thus, temporary inhibition of PKR is likely to be tolerable.

The foregoing observations encouraged us to study the course of Mtb infection in PKR-deficient mice. PKR-deficient mice sustained lower Mtb burdens and had less pulmonary pathology than wild type mice. In seeking an explanation, we discovered that PKR-deficient macrophages underwent more apoptosis than wild type macrophages when infected with Mtb in vivo and in vitro, and produced more iNOS and TNF-alpha in response to IFN-gamma. In wild type macrophages, IFNγ activated PKR. PKR in turn mediated induction of the macrophage-deactivating factor IL10. At the low levels induced by IFN-gamma, IL10 tempered, without preventing, IFN-gamma-dependent macrophage activation. Thus, in the absence of PKR, macrophages were more fully activated. These findings raise the possibility that a PKR inhibitor could boost host immunity to serve as an adjunctive treatment for tuberculosis and perhaps other infections.

## Results

### Impact of PKR Deficiency on Tuberculosis

**Figure 1:**
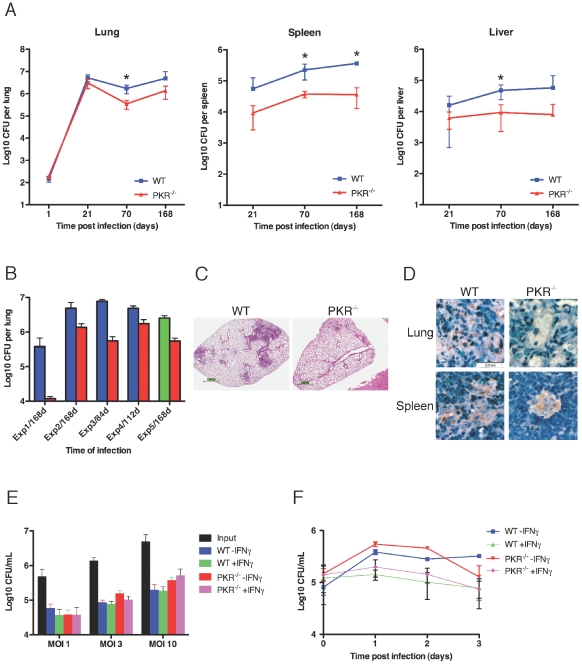
Course of Mtb infection in PKR-deficient and wild type mice. (A) Time course of burden of colony-forming units (CFU) in lung, liver and spleen over 24 weeks following infection by inhalation of an average of 30 CFU by wild type mice and 37 CFU by PKR-deficient mice. Results are means ± SD for 5 mice per time point. Asterisks mark time points at which the differences had p values≤0.01 by Student's t test. (B) Mtb burdens (mean CFU ± SEM for 4–6 mice per strain) in lungs of mice from 5 independent experiments at the latest time point post-infection evaluated in each experiment. Red bars: PKR^−/−^ mice. Blue bars: C57LB/6 wild type controls. Green bar: 129S1/SvImJ wild type controls. The initial bacterial burdens were comparable between mouse strains within each experiment as assessed by the following CFU counts 24 hours post infection (pairs are means for wild type mice followed by PKR-deficient mice in individual experiments in the order depicted in the figure): 30, 37; 15, 20; 24, 22; 488, 468; 40, 45. The unusually low CFU seen in the first experiment are depicted with reference to an expanded Y-axis. (C and D) Histopathology (C) and acid-fast staining of Mtb (D) in lungs from Mtb-infected wild type and PKR^−/−^ mice at day 168 from a representative experiment. (E and F) Comparable uptake and control of Mtb by PKR-deficient and wild type macrophages in vitro. (E) Uptake of Mtb 4 hours after addition at the indicated MOI, with and without exposure of macrophages to IFN-gamma (10 ng/mL) overnight in advance of infection. (F) Intracellular growth of Mtb (MOI = 3) with and without exposure of macrophages to IFN-gamma (10 ng/mL) overnight in advance of infection. With the other MOIs tested (1 and 10), there was likewise no significant difference in the numbers of CFU in PKR-deficient and wild type macrophages over the 3 days studied.

To determine the impact of PKR deficiency on tuberculosis, we compared the bacterial burden in Mtb-infected wild type and PKR-deficient mice up to 168 days after low-dose infection by aerosol (Figure 1A). The course of infection in the lung was indistinguishable between the two strains through the first 21 days, corresponding to the phase of exponential replication that precedes the onset of a full adaptive immune response. By days 70 and 168, during the chronic phase of the infection, the bacterial burden in lungs, liver and spleen of the PKR-deficient strain was 4- to 10-fold lower than in the wild type (Figure 1A). In 5 independent experiments—4 using C57BL/6J mice and one using 129S1/SvImJ mice as the wild type strain– the CFU in lungs of PKR-deficient mice were 2.7-, 3.6-, 13.9-, 41.8-fold and 4.6-fold (mean, 13-fold) lower than in wild type mice at the latest time point studied in each experiment (Days 84, 112 or 168) (Figure 1B). The improved control of Mtb in PKR-deficient mice was statistically significant (p<0.001 by Wilcoxon's matched pair test).

We assessed the extent of pathology in Mtb-infected, PKR-deficient mice compared to that in wild type mice to see if the reduced bacterial burden might have resulted from recruitment of larger numbers of more destructive immune and inflammatory cells or, conversely, may have resulted in fewer immune or inflammatory cells being attracted or retained. We prepared 5 sections evenly spaced through each fixed lung from all mice 24 weeks after infection with Mtb in the experiment shown in Figure 1A. Two observers assigned each hemotoxylin and eosin-stained section a score as follows: 0 = no areas of diffuse or consolidated infiltration (normal histology); 1 = 1–3 areas of diffuse infiltration or consolidation by cells with the morphology of monocytes, macrophages, epithelioid macrophages or lymphocytes, each such area having a diameter <10% of the long axis of the lung section; 2 = 1–2 areas of consolidation each with a diameter >10% of the long axis of the lung section (“large” consolidation); 3 = 3–5 areas of large consolidation; 4 = 4–15 areas of large consolidation; 5 = >15 areas of large consolidation. In the wild type mice, the scores averaged 2.7±0.3 (mean ± SE). In the PKR-deficient mice, the scores averaged 1.4±0.2. The difference was statistically significant (p<0.05 by Mann Whitney's two-tailed U-test). Figure 1C shows sections that scored close to the mean for each genotype. PKR-deficient lungs also revealed fewer Mtb visible by acid-fast staining (Figure 1D).

The phenotype of improved control of Mtb infection by PKR-deficient mice over many months during the chronic phase of the infection was not recapitulated in macrophages that were differentiated from bone marrow in vitro and studied in isolation for several days. In vitro, the extent of uptake of Mtb over 4 hours and its subsequent intracellular growth without IFN-gamma or lack of growth in the presence of IFN-gamma over 3 days were indistinguishable in macrophages derived from wild type and PKR-deficient mice (Figures 1E and 1F). This suggested that in vitro studies on such macrophages could reveal only limited aspects of phenomena involving multiple cell types, physiologic environments and long periods of time in vivo. At the same time, however, these findings suggested that comparisons of macrophages in vitro from wild type and PKR-deficient mice would not be confounded by dissimilar loads of Mtb.

### Impact of PKR Deficiency on Apoptosis of Mtb-Infected Macrophages

**Figure 2:**
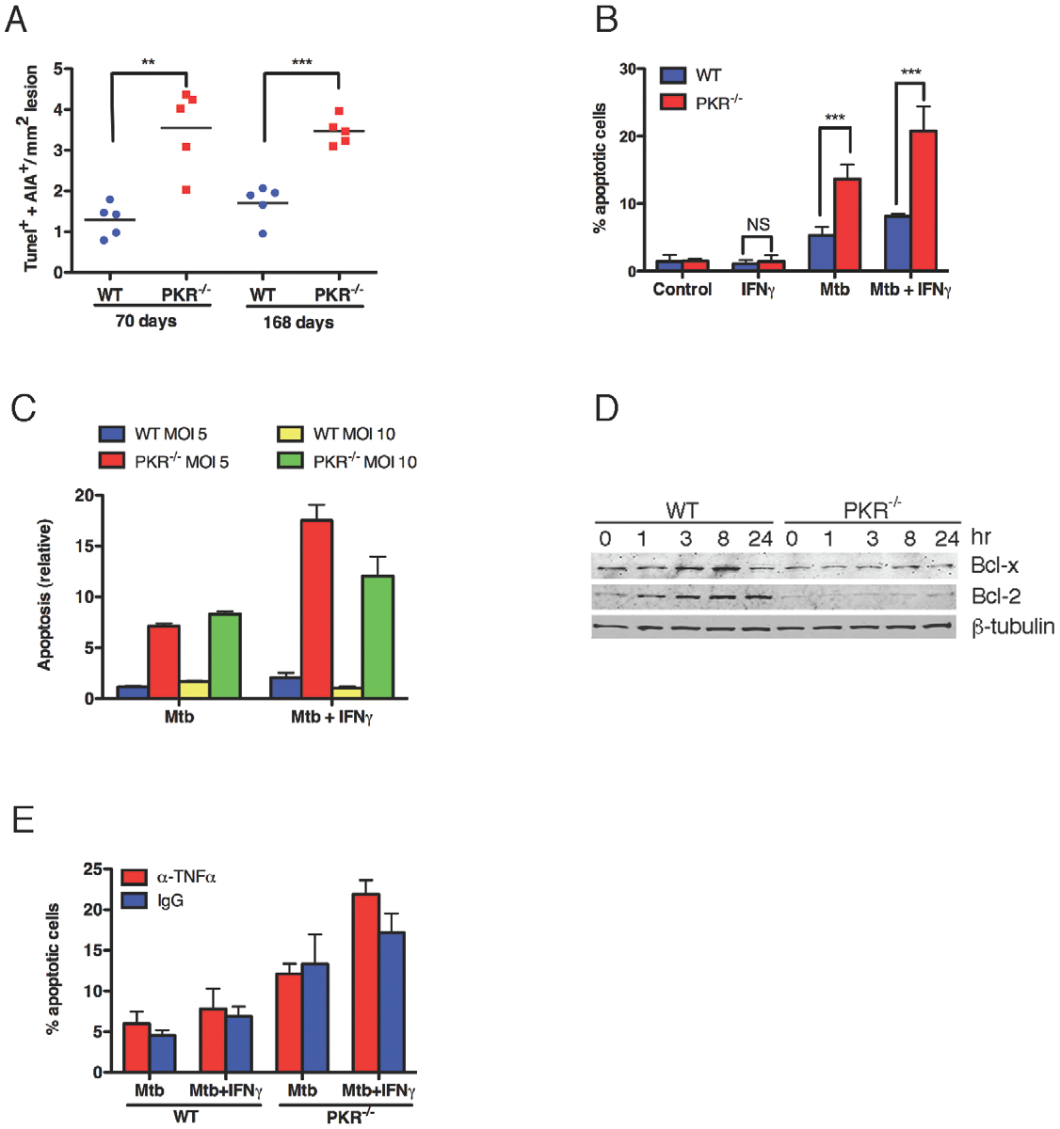
Enhanced apoptosis of the PKR-deficient macrophages. (A) Apoptosis of macrophages in vivo. After 70 or 168 days of infection, sections of lungs from 5 WT and 5 PKR^−/−^ mice were stained by TUNEL and with an anti-macrophage antibody, AIA [58]. TUNEL-positive macrophages were counted in 4 microscopic fields per lung in areas of comparable histopathology such that approximately 20–40 TUNEL-positive cells were evaluated per mouse. **, p<0.005, ***; p<0.0005 (unpaired *t* test). (B) Apoptosis of macrophages in vitro assessed by TUNEL. Macrophages (5×10^4^) from wild type and PKR^−/−^ mice were incubated with or without IFNγ (10 ng/mL) overnight and then infected with Mtb at MOI 10. After 24 h, apoptosis was assayed by counting the proportion of TUNEL-positive macrophages in 4 microscopic fields per well. Control, no Mtb. ***, p≤0.0005 (unpaired *t* test). NS, not significant. (C) Apoptosis of macrophages in vitro assessed by ELISA for cytoplasmic histone-associated mono- and oligo-nucleosomes. Macrophages were infected as in (B). The relative extent of apoptosis is presented as a ratio of absorbance values from infected macrophages to those from uninfected macrophages. In (B) and (C), results are means ± SD for 3 replicates in one experiment representative of 2. (D) Western blot for apoptosis-inhibitory proteins at the indicated times after infection of macrophages with Mtb at MOI = 1. Immunoblot for β-tubulin served as a loading control. (E) Effect of anti-TNF-alpha on apoptosis of primary macrophages in response to Mtb infection. Macrophages from WT and PKR^−/−^ mice were incubated with or without IFN-gamma (10 ng/mL) overnight and then treated with anti–TNF-alpha IgG (10 µg/mL) or isotype-matched irrelevant IgG just before the infection with Mtb at MOI 10 for 24 hr. Apoptosis was assayed by staining with TUNEL and quantified as the percentage of TUNEL positive cells among DAPI positive cells. Data are means ± SD for 4 fields of cells for each condition.

Among the major processes by which macrophages control Mtb are apoptosis [28], [29] and the expression of iNOS and TNF-alpha. To test for an apoptotic phenotype in vivo, lung sections from Mtb-infected wild type and PKR-deficient mice were stained by TUNEL to identify apoptotic cells and with an antibody to identify macrophages. By day 70, when Mtb infection was well advanced, the proportion of apoptotic macrophages was 2.8-fold higher in the lungs of PKR-deficient mice than wild type. The pro-apoptotic phenotype persisted through at least day 168, with a 2.1-fold higher proportion of apoptotic macrophages in the PKR-deficient mice (Figure 2A). We confirmed the pro-apoptotic phenotype of Mtb-infected, PKR-deficient macrophages using bone marrow derived macrophages in vitro, as judged both by TUNEL staining (Figure 2B) and by an ELISA for cytoplasmic histone-associated DNA (Figure 2C). The greater apoptotic response of PKR-deficient macrophages than wild type macrophages to Mtb infection was associated with markedly decreased expression of the anti-apoptotic proteins Bcl-x and Bcl-2 (Figure 2D). Because it will be shown below that PKR-deficient macrophages produce more TNF than wild type macrophages in response to IFN-gamma, it was important to test if the exaggerated apoptotic phenotype of PKR-deficient, Mtb-infected macrophages might be influenced by their production of TNF-alpha. However, the increased level of apoptosis persisted in the presence of neutralizing antibody to TNF-alpha (Figure 2E).

### Impact of PKR Deficiency on Production of Reactive Nitrogen Intermediates

**Figure 3:**
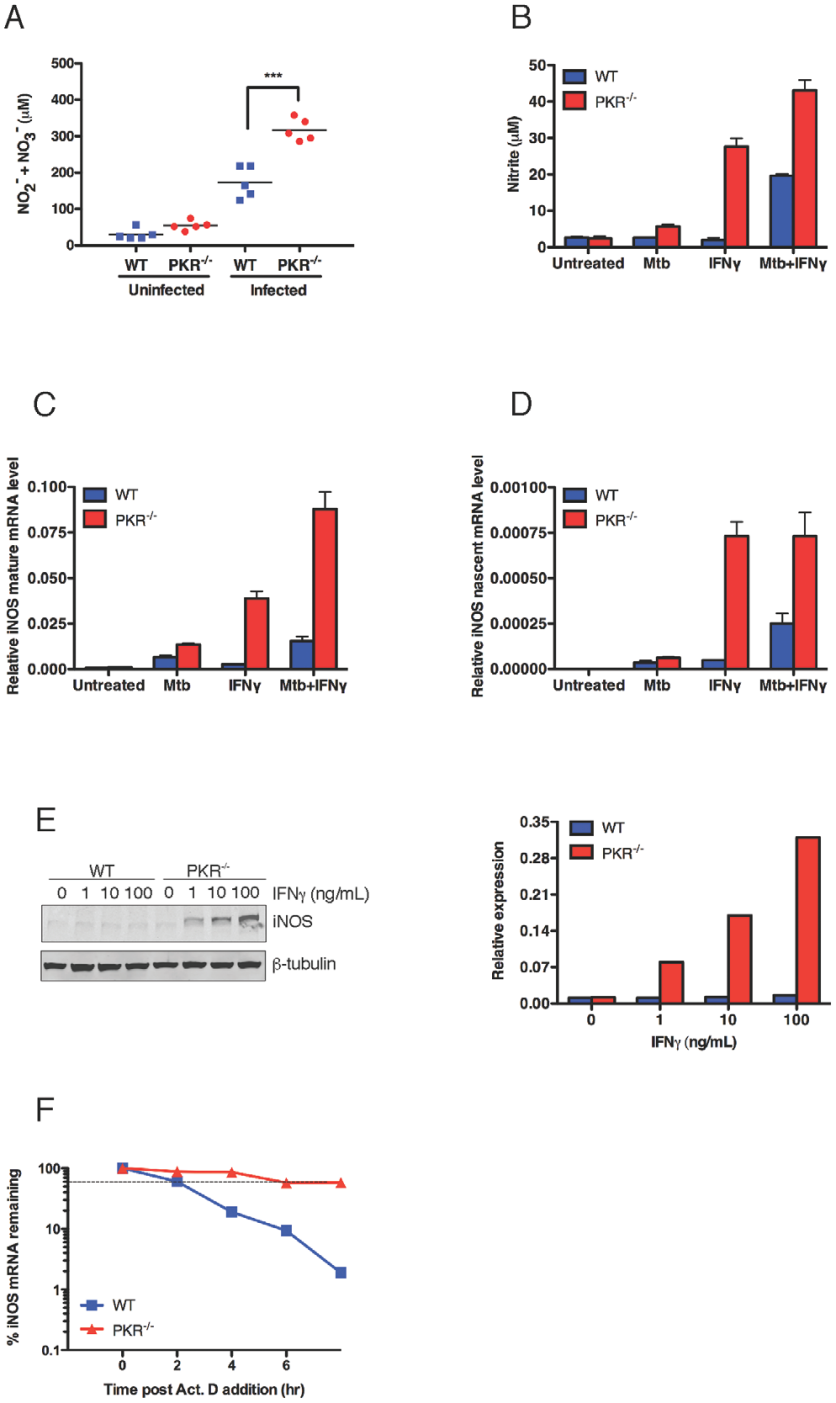
Enhanced expression of iNOS in PKR-deficient mice and macrophages in response to Mtb and IFN-gamma. (A) Nitrite plus nitrate in sera from Mtb-infected wild type and PKR-deficient mice. Nitrite and nitrate in serum from Mtb-infected mice at day 168 were measured by the Griess reaction after nitrate was reduced to nitrite as described [30]. Means ± SD of 4–5 mice in one experiment representative of two. ***, p<0.0005 (unpaired *t* test). (B) Secretion of nitrite by macropahges in vitro. Macrophages (5×10^5^) were treated with Mtb (MOI 3), IFN-gamma (10 ng/mL), or pre-treated with IFN-gamma (10 ng/mL) overnight followed by infection with Mtb (MOI 3) for 24 h. Nitrite in the supernatant was determined by the Griess reaction. Means ± SD of triplicates. (C and D) Mature and nascent iNOS transcripts. Macrophages were incubated as in (B) for 3 h. qRT-PCR signals for iNOS were normalized to GAPDH. Means ± SD of triplicates. In (B), (C) and (D), results are from one of two similar experiments. Results were similar in additional experiments at MOI's of 0.3, 1 and 3 and IFN-gamma concentrations of 1, 10 and 100 ng/mL. (E) Western blot (left) and its densitometric assessment (right) for iNOS in macrophages incubated with the indicated concentrations of IFN-gamma for 24 h. Immunoblot for beta-tubulin served as a loading control. (F) Impact of PKR deficiency on stability of iNOS mRNA. Macrophages were treated with IFN-gamma (10 ng/mL) followed 3 h later by actinomycin D (10 micrograms/mL). RNA was extracted at the indicated times for RT-PCR and normalized to GAPDH.

Because control of tuberculosis in mice depends on expression of iNOS [30], we tested whether improved control in PKR-deficient mice reflected enhanced generation of reactive nitrogen intermediates (RNI). To assess this at the level of product formation, we measured nitrite and nitrate, the accumulating autoxidation products of NO, in the serum of Mtb-infected mice at day 168 post-infection. In two independent experiments, the level of these RNI was about twice as high in serum from PKR-deficient mice as from wild type mice (p<0.0005 by unpaired *t* test) (Figure 3A), despite the lower burden of bacteria to serve as a co-inducer of iNOS expression [11].

We used bone marrow derived macrophages in vitro to investigate the mechanisms for the in vivo phenotype of Mtb-infected, PKR-deficient mice. As noted, induction of iNOS in mouse macrophages in vitro usually requires two signals—a cytokine such as IFN-gamma and a microbe or microbial product [11]. This was the case with wild type macrophages in the present experiments. PKR-deficient macrophages responded to the combination of Mtb and IFN-gamma with higher levels of unspliced iNOS transcripts, mature iNOS transcripts and iNOS protein than wild type macrophages (Figure 3B–D). Strikingly, however, PKR-deficient but not wild type macrophages also expressed iNOS in response to IFN-gamma alone (Figure 3D and E). Induction of iNOS by IFN-gamma as sole stimulus in PKR-deficient macrophages was both transcriptional (Figure 3D) and post-transcriptional (Figure 3F). The estimated *t*_1/2_ of iNOS mRNA in IFN-gamma -treated WT macrophages was 2 hr, while the *t*_1/2_ of iNOS mRNA in PKR^−/−^ macrophages was prolonged to 6–8 hr. Thus, PKR suppressed both the transcription and the stability of iNOS mRNA in IFN-gamma –treated macrophages.

### Impact of PKR Deficiency on Production of TNF-alpha

**Figure 4:**
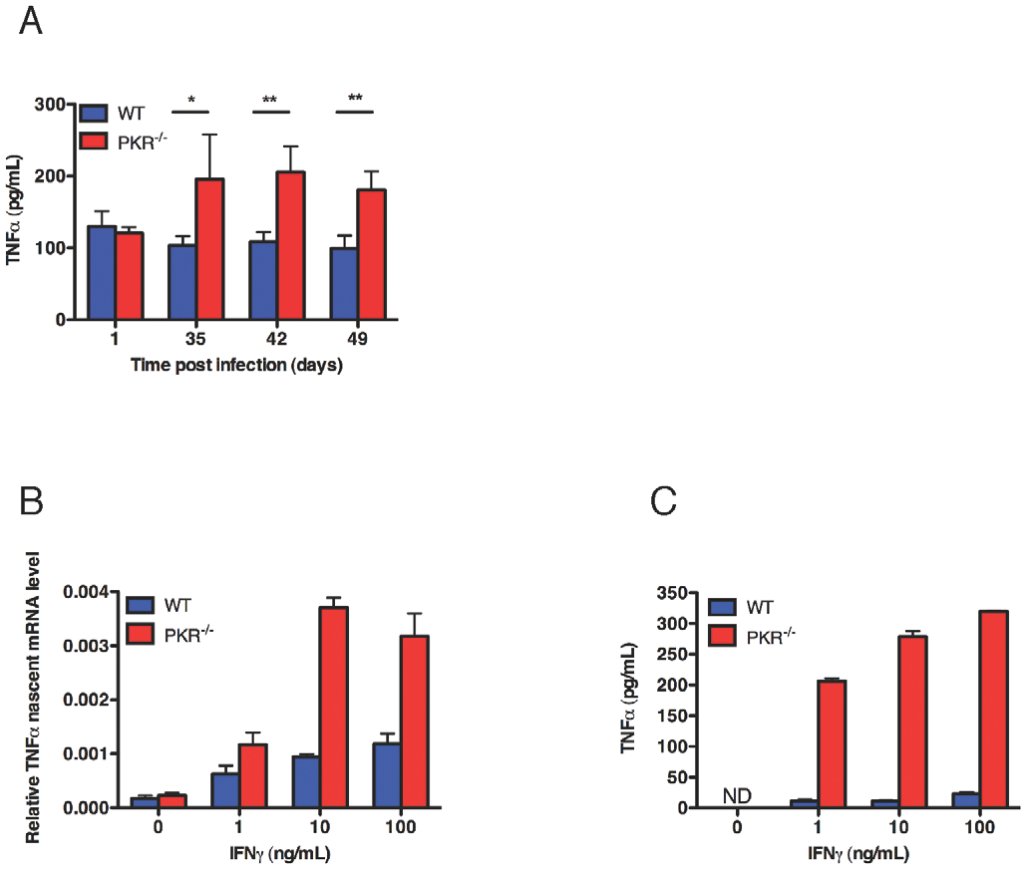
Enhanced expression of TNF-alpha in PKR-deficient mice and macrophages. (A) Levels of TNF-alpha in lung homogenates. TNF-alpha in lung homogenates from Mtb-infected mice at indicated time was measured by ELISA. Means ± SD for 4 mice per strain from one experiment representative of four. *, p<0.05, **, p<0.005, (unpaired t test). (B) Nascent TNF-alpha transcripts. Macrophages (5×10^5^) were incubated with the indicated concentrations of IFN-gamma for 3 h. qRT-PCR signals for TNF-alpha were normalized to GAPDH. Means ± SD of triplicates. (C) Secretion of TNF-alpha. Macrophages (5×10^5^) were incubated with the indicated concentrations of IFN-gamma for 48 h. Supernatant was collected for TNF-alpha production by ELISA. Means ± SD of triplicates. In (B) and (C), results are from one of two similar experiments.

Two cytokines are known to be critical for humans to control tuberculosis: IFN-gamma, as judged by the predisposition to mycobacterial infection in people with genetic deficiency states in proteins involved in IFN-gamma production and signaling [31], and TNF-alpha, as revealed by the risk of reactivation of latent tuberculosis infection in people given therapeutic agents that neutralize TNF-alpha in the treatment of inflammatory diseases [32]. Accordingly, we tested the production of TNF-alpoha in vivo and in vitro. In four of five independent experiments in Mtb-infected mice, levels of TNF-alpha were significantly higher in homogenates of the PKR-deficient lungs than in wild type lungs, despite the lower bacterial burden in the PKR-deficient lungs (Figure 4A). In vitro, IFN-gamma alone was sufficient to induce TNF-alpha at the transcriptional level in PKR-deficient but not wild type macrophages (Figure 4B, C).

### Role of IL-10 in PKR-mediated Inhibition of Macrophage Activation

**Figure 5:**
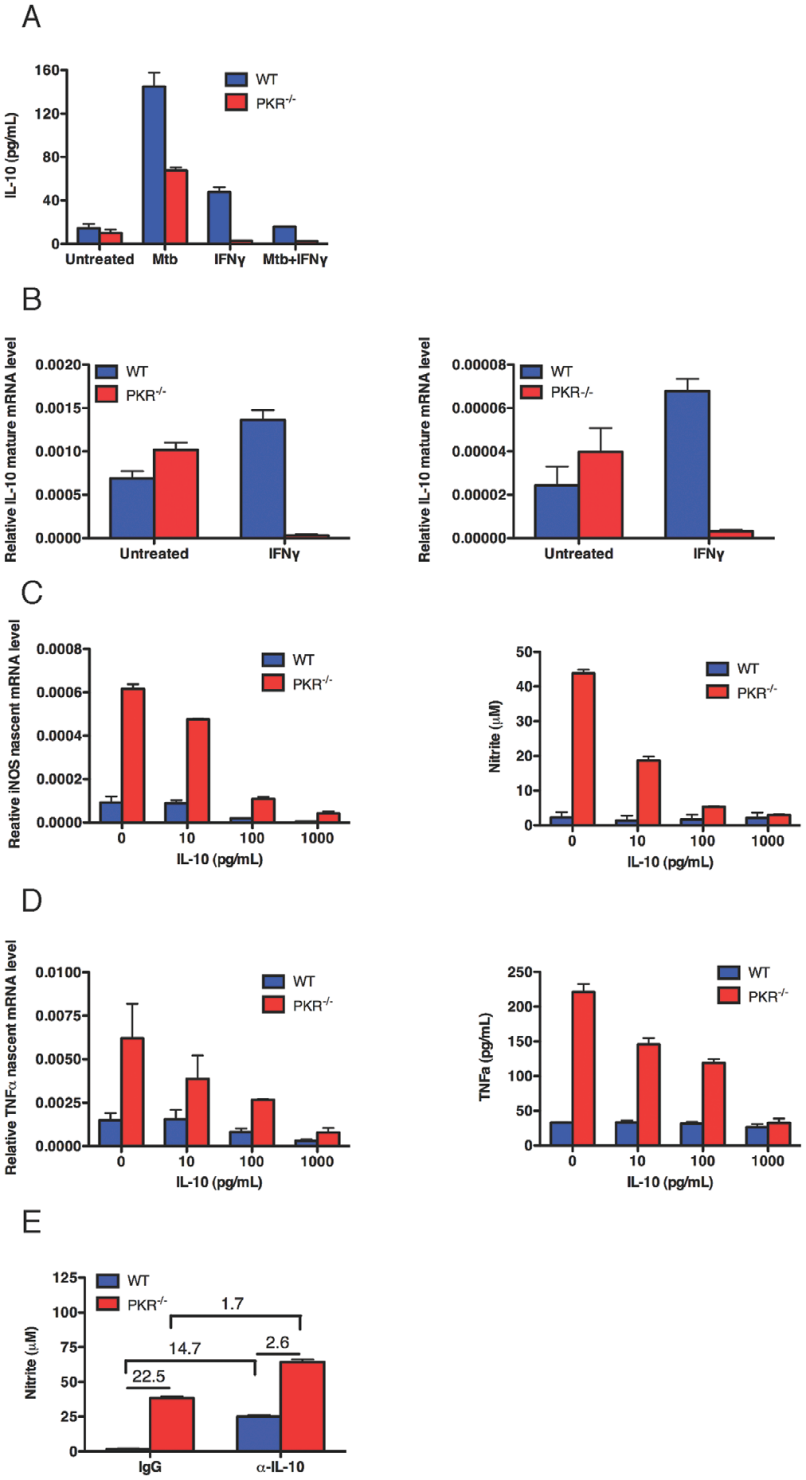
Induction of IL-10 by Mtb and IFN-gamma and its impact on iNOS and TNF-alpha. (A) Secretion of IL-10. Macrophages (5×10^5^) were treated with Mtb (MOI 3), IFN-gamma (10 ng/mL), or pre-treated with IFN-gamma (10 ng/mL) overnight followed by infection with Mtb (MOI 3) for 24 h. Supernatant was collected for IL-10 production by ELISA. Results were similar in additional experiments with MOIs of 0.3, 1 and 3 and IFN-gamma concentrations of 1, 10 and 100 ng/mL. (B) Induction of mature and nascent IL-10 transcripts. Macrophages (5×10^5^) were treated with IFN-gamma (10 ng/mL) for 3 h. qRT-PCR signals for IL-10 were normalized to GAPDH. Results were similar with IFN-gamma concentrations of 1, 10 and 100 ng/mL. (C) Effect of IL-10 on nascent iNOS transcripts and secretion of nitrite. Left panel, macrophages (5×10^5^) were treated with IL-10 for 3 h after the addition of IFN-gamma 0 ng/mL. qRT-PCR signals for iNOS were normalized to GAPDH. Right panel, macrophages (5×10^5^) were treated with IL-10 for 48 h after the addition of IFN-gamma 0 ng/mL. Nitrite in the supernatant was measured by the Griess reaction. (D) Effect of IL10 on nascent TNF-alpha transcripts and TNF-alpha. Left panel, macrophages (5×10^5^) were treated with IL-10 for 3 h after the addition of IFN-gamma 0 ng/mL. qRT-PCR signals for TNF-alpha were normalized to GAPDH. Right panel, macrophages (5×10^5^) were treated with IL-10 for 48 h after the addition of IFN-gamma 0 ng/mL. TNF-alpha in the supernatant was measured by ELISA. (E) Effect of neutralizing anti-IL-10 antibody (10 µg/mL) on release of nitrite 48 h after addition of IFN-gamma (10 ng/mL). Results in (A) to (F) are means ± SD from individual experiments each with 3 replicates that are representative of at least 2 independent experiments.

PKR has been reported to mediate IL10 induction in macrophages in response to dsRNA, LPS and Sendai virus [24]. When produced by a population of macrophages, IL10 can potently suppress the expression of iNOS and TNF-alpha in the same population [14], [15]. Accordingly, we measured IL10 production by Mtb-infected bone marrow-derived macrophages. Mtb infection induced about twice as much IL10 in wild type as in PKR-deficient macrophages (Figure 5A). IFN-gamma is well known to reduce production of IL10, not induce it. As expected, IFN-gamma reduced the levels of IL10 induced by Mtb (Figure 5A). In contrast, to our surprise, IFN-gamma alone induced low levels of IL10 in uninfected wild type macrophages, and not in PKR-deficient cells (Figure 5A). Induction of IL10 by IFN-gamma in wild type macrophages was manifest by increases in nascent and mature transcripts as well as secreted protein (Figure 5B).

When IL10 was added to IFN-gamma-treated PKR-deficient macrophages, nascent iNOS transcripts, nitrite, nascent TNF-alpha transcripts and TNF-alpha protein were all reduced to the level of wild type cells (Figure 5C–D). The amounts of reagent IL10 required to phenocopy wild-type behavior in IFN-gamma-treated PKR-deficient macrophages were higher than those released by wild type macrophages in response to IFN-gamma. This may reflect lower specific activity of the recombinant protein than the natural protein, or the involvement of an additional cytokine besides IL10. Conversely, when anti-IL10 neutralizing antibody was added to IFN-gamma-treated wild type macrophages, their production of nitrite rose 22.5-fold, while production of nitrite rose only 2.6-fold in PKR-deficient macrophages (Figure 5E). Thus, PKR represses IFN-gamma-dependent macrophage activation by mediating compensatory IL10 production.

### Effect of a PKR Inhibitor on Macrophage Activation in vitro

**Figure 6:**
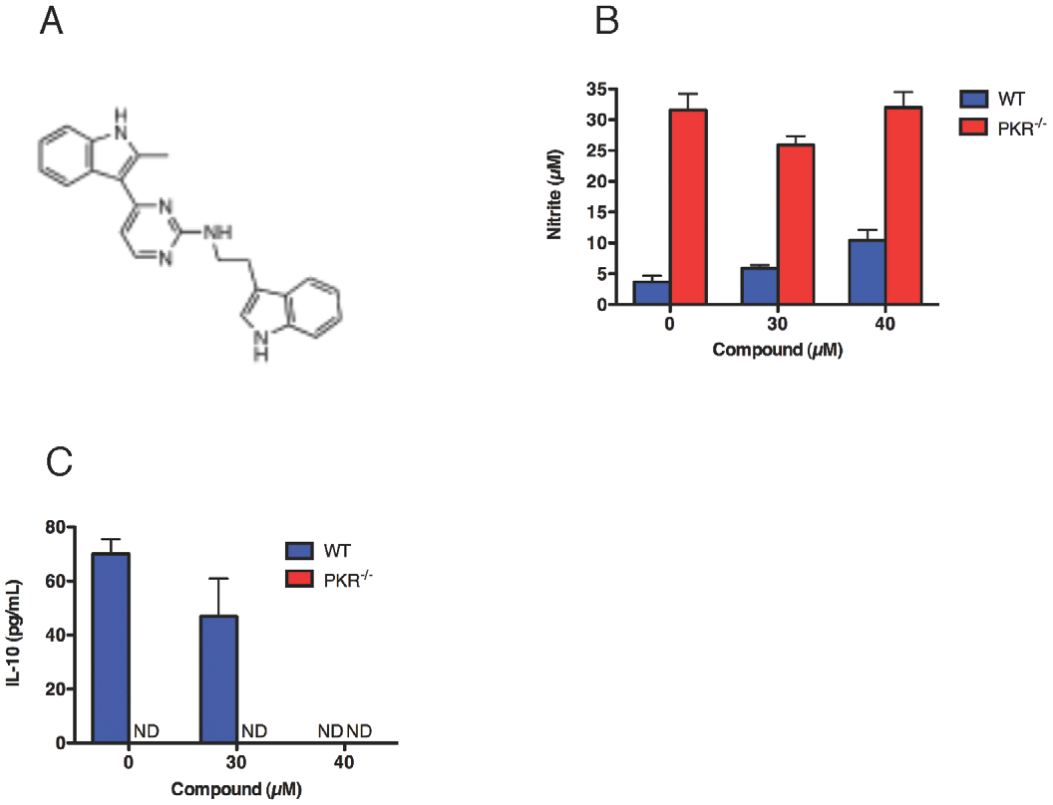
A PKR inhibitor partially phenocopies the effect of PKR deficiency on release of nitrite and IL10 in response to IFN-gamma. (A) Structure of the PKR inhibitor *N*-(2(1*H*-indol-3-yl)ethyl)-4-(2-methyl-*1H*-indol-3-yl)pyrimin-2-amine. (B) Impact of the inhibitor on nitrite release 48 h after addition of IFN-gamma (10 ng/mL) to wild type macrophages. (C) Impact on IL10 secretion 48 h after addition of IFN-gamma (10 ng/mL) to wild type macrophages. (B) and (C) are means ± SD for 3 replicates in one of 3 similar experiments.

A novel PKR inhibitor was recently identified that can inhibit the enzyme within intact macrophages at mid-micromolar concentrations [33]. When applied to wild type macrophages, the inhibitor, *N*-(2(1*H*-indol-3-yl)ethyl)-4-(2-methyl-*1H*-indol-3-yl)pyrimin-2-amine (Figure 6A), partially phenocopied PKR deficiency in promoting nitrite release (Figure 6B) and suppressing IL10 release (Figure 6C) in response to IFN-gamma. Both promotion of nitrite release and the morphologic integrity of the macrophages (not shown) demonstrated that the inhibitor was not toxic under the conditions tested. These observations suggested that PKR's role in promoting IL10 release and suppressing macrophage activation depended on its enzyme activity.

### Activation of PKR by IFN-gamma

**Figure 7:**
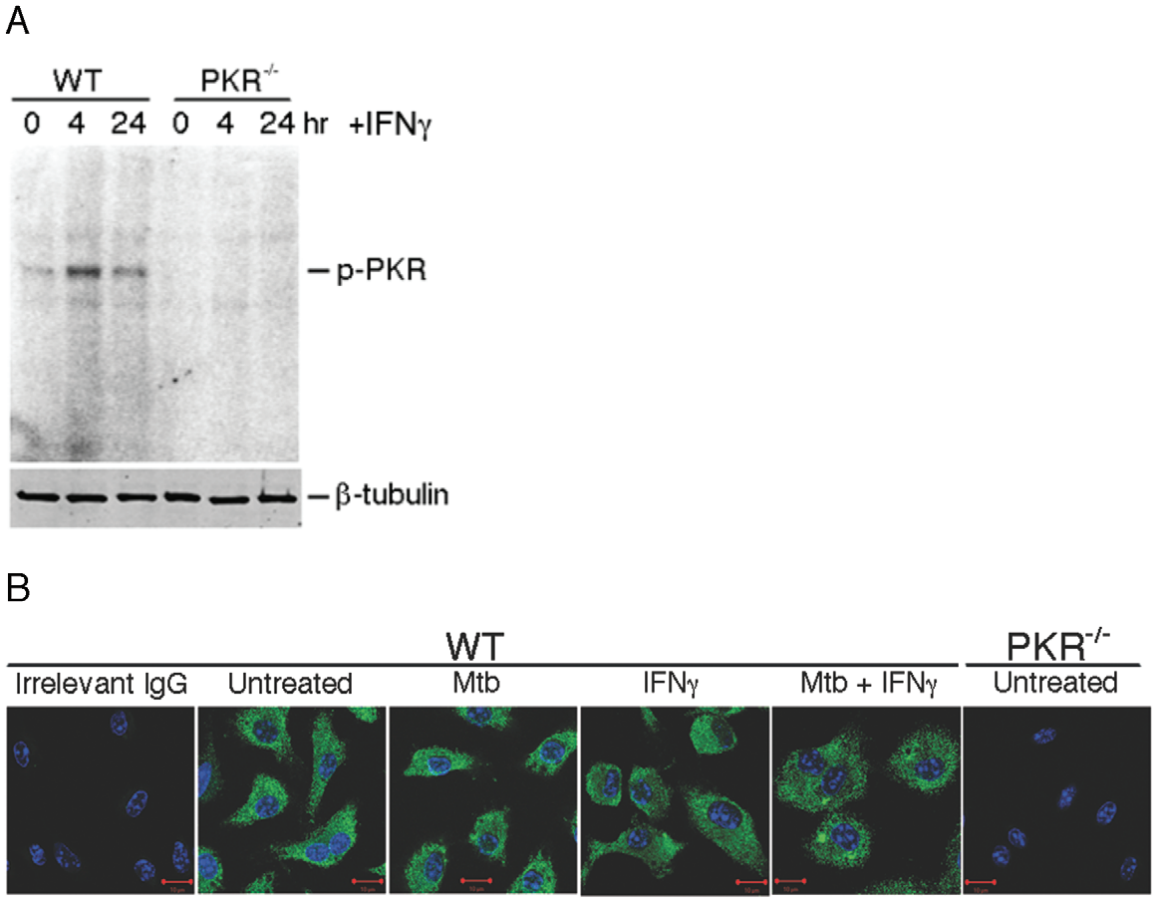
Activation of PKR by IFN-gamma. (A) Autophosphorylation of PKR in macrophages at the indicated times after addition of IFN-gamma (10 ng/mL). A duplicate gel western blotted with antibody to beta-tubulin served as a loading control. One of four similar experiments. (B) Immunofluorescent localization of PKR in wild type macrophages with and without Mtb infection and/or IFN-gamma treatment. Macrophages from wild type and PKR^−/−^ mice were incubated with or without IFN-gamma (10 ng/mL) at 37°C overnight and then infected with Mtb at MOI 10 for 24 h. Macrophages were fixed with 4% *p*-formaldehyde for 2 h and immunofluorescent staining for PKR (green) and DNA (DAPI; blue) was conducted. Images were recorded by confocal microscopy.

There has been little evidence that IFN-gamma activates PKR [34], but our results strongly suggested that this is the case in macrophages. In fact, as shown in Figure 7A, PKR was rapidly phosphorylated in IFN-gamma-treated macrophages. Because PKR affected IFN-gamma-induced transcription of iNOS, TNF-alpha and IL10, we considered that IFN-gamma might trigger the translocation of PKR to the nucleus, where it could phosphorylate proteins participating in transcriptional control. However, confocal immunofluorescence microscopy demonstrated that all detectable PKR was cytosolic and remained so with and without exposure of the macrophages to IFN-gamma and/or infection by Mtb. Specificity of the staining was confirmed by its absence in PKR-deficient cells (Figure 7B). Thus, PKR probably acts in the cytosol to mediate aspects of IFN-gamma signaling. We detected no differences in the phosphorylation of ERK1/2, p38, STAT1 or STAT3 in response to IFN-gamma in wild type and PKR-deficient macrophages (Figure S1). However, the binding of STAT1 to an IFN-gamma-activated site (GAS) from the iNOS promoter was diminished in extracts from the nuclei of IFN-gamma-activated, PKR-deficient macrophages compared to wild type (Figure S2).

## Discussion

PKR deficiency led to reduced bacterial burden and reduced pathology in mice with tuberculosis. Macrophages in the lungs of PKR-deficient mice showed more extensive apoptosis than in wild type mice and PKR-deficient mice accumulated higher levels of RNI in their blood. In vitro, PKR-deficient macrophages underwent more extensive activation than wild type macrophages in response to IFN-gamma alone or the combination of exposure to IFN-gamma and infection with Mtb. A major mechanism at work was the IFN-gamma-mediated activation of PKR in wild type macrophages, leading to induction of IL10. IL10 acted to restrain the extent to which macrophages produced iNOS and TNF-alpha, two critical mediators of host control of tuberculosis.

Our studies do not establish to what extent the IL10-dependent mechanisms uncovered in vitro account for the in vivo phenotype of PKR-deficient mice during Mtb infection. However, the observations reported here do implicate the enzymatic action of PKR, because a PKR inhibitor phenocopied key aspects of the PKR-deficient phenotype in vitro. Very few PKR inhibitors have been reported and none have undergone the structural optimization or mechanistic and pharmaceutical characterization that could qualify them for testing in mice [33]. However, the present observations suggest that PKR inhibitors with the requisite potency, selectivity and bioavailability should be developed for tests of efficacy in experimental tuberculosis. The candidacy of PKR as a therapeutic target has been suggested in a number of other diseases, including neurodegeneration, obesity, diabetes and influenza [26], [35], [36], [37], [38], [39].

The enhanced apoptosis of PKR-deficient macrophages upon Mtb infection may have contributed substantially to improved control of the infection [40]. Genetic evidence in both the host [41] and the pathogen [42], [43] supports the importance of apoptosis as a means of limiting Mtb's virulence and promoting its immunogenicity. PKR has different effects on apoptosis and activation in various cells and situations [23]. PKR was required for mouse macrophages to undergo apoptosis in response to the TLR4 agonists LPS, *B. anthracis*, *S. enterica* var. *Typhimurium* and *Y. pseudotuberculosis*, particularly when p38 was pharmacologically blocked [44]. However, Mtb predominantly stimulates TLR2 [45], [46] rather than TLR4, and p38 inhibitors were not used in our experiments. These differences may help explain why we found that PKR restrained, rather than promoted, apoptosis in Mtb-infected macrophages. In the study of Hsu et al., iNOS induction by LPS was impaired in PKR-deficient macrophages [44]. Similarly, neither LPS nor poly-IC could induce iNOS in PKR-deficient mouse embryonic fibroblasts [47]. In our hands, when used as a sole stimulus, LPS alone and IFN-gamma alone each induce little or no iNOS in wild type macrophages. In contrast, IFN-gamma used as sole stimulus induced abundant iNOS in macrophages that lacked PKR. Moreover, the combination of IFN-gamma and Mtb induced more iNOS in PKR-deficient macrophages than in wild type cells. Thus, depending on the stimuli, PKR can either promote or restrain classical macrophage activation. With IFN-gamma and Mtb as stimuli, the predominant action of PKR appears to be to restrain macrophage activation by enabling the induction of counter-regulatory IL10.

That PKR can help mediate IFN-gamma signaling has been suggested, but to our knowledge, this had not previously been demonstrated by altered expression of an IFNγ-induced protein in the absence of another stimulus. For example, compared to wild type, PKR^−/−^ embryonic fibroblasts responded to IFN-gamma with less phosphorylation of p38 [48] and STAT1 [49] and with less activation of NF-kappaB [50] and IRF1 [47] in electrophoretic mobility shift assays, but effects on protein expression were not described. In macrophages, we did not detect an effect of PKR on phosphorylation of p38 or STAT1 in response to IFN-gamma, and IFN-gamma did not appear to activate NF-kappaB (not shown).

PKR has been reported to mediate IL10 induction in macrophages in response to dsRNA, LPS and Sendai virus [24]. However, it has apparently not been appreciated that IFN-gamma can induce low-level IL10 expression in macrophages, or that PKR is required for this response. To the contrary, it is well established that IFN-gamma suppresses IL10 expression in response to another agonist [51], [52]. That IFN-gamma can have both actions is consistent with emerging evidence that pro-inflammatory stimuli often induce their own antagonists [53]. The ability of T cell clones to produce IFN-gamma and IL10 simultaneously is well accepted as a mechanism by which the host can both induce and restrain immune responses [54]. The ability of macrophages to respond to IFN-gamma by making small amounts of IL10 can be seen in the same light.

Specifically, we found that IFN-gamma blunted the induction of IL0 by Mtb in *infected* macrophages, yet *induced* a small amount of IL10 by itself in *uninfected* macrophages. Most macrophages in tuberculosis lesions do not contain Mtb, yet they are exposed to the same cytokines as the macrophages that are infected. Immunopathology would be reduced if the uninfected macrophages were to undergo less activation than the infected ones. Induction of low levels of IL10 by IFN-gamma in uninfected macrophages could serve to temper, without preventing, the pro-inflammatory effects of IFN-gamma. The evolved function of such a circuit is presumably to reduce immunopathology during infection. However, in the studies described here, deletion of PKR apparently led to enough of a reduction in bacterial burden to avoid the risk of enhanced immunopathology.

PKR was not identified as an Mtb-related CGEP in RNAi screens that targeted all kinases [6] or all genes [7] in macrophage cell lines. In retrospect, this is not surprising, because we found that PKR deficiency had no effect on the ability of macrophages to take up Mtb or restrain its growth in vitro (data not shown). It is not clear why PKR deficiency was associated with reduced growth of Mtb in vivo but not in vitro. In general, macrophage control of Mtb in vitro appears to be far less effective than in the host [55].

In conclusion, to our knowledge, the present report appears to be the first to identify a host enzyme whose absence is beneficial during infection of the host with Mtb.

## Materials and Methods

### Ethics Statement

Mice were studied under a protocol (#0512-422) approved by the Weill Cornell Medical College Institutional Animal Care and Use Committee.

### Mtb

We grew Mtb strain H37Rv to early log phase in Middlebrook 7H9 broth (BD Biosciences) with BBL Middlebrook OADC Enrichment (Becton Dickinson) and 0.05% (vol/vol) Tween 80 (Sigma-Aldrich). Predominantly single-cell suspensions were collected by centrifuging twice at 125 g, resuspending in PBS with 0.05% Tween 80 and using the supernate from 10 min centrifugation at 125 g to infect macrophages or mice.

### Mice and Macrophages

Young adult female C57BL/6 and 129S1/SvImJ mice were from Jackson Laboratories. PKR^−/−^ mice were interbred locally from founders kindly provided by Prof. Charles Weissmann (University of Zurich) [56]. PKR was undetectable in primary bone marrow-derived macrophages from these mice, as judged by western blot and poly IC-stimulated kinase activity (Figure S3). Tail biopsies were lysed in 500 µl 50 mM Tris-HCl (pH 8.0), 100 mM EDTA, 100 mM NaCl, 1% SDS, and 0.1 mg/mL proteinase K at 56°C overnight. DNA was precipitated with one volume of isopropanol, washed with cold 70% ethanol and 100% ethanol and dissolved in TE buffer (10 mM Tris-HCl, 1 mM EDTA, pH 8.0). PCR primers complementary to the neomycin cassette NeoF (5′-3′) CAGGTAGCCGGATCAAGCGTATGC and NeoR (5′-3′) CCTGTCCGGTGCCCTGAATGAACT generated a 200-bp fragment. Primers corresponding to PKR intron 1 and intron 3, PkrF-1 (5′-3′) AGCGGCCCTGTCTCCTGTTCT and PkrR-1 (5′-3′) TGCTGGGGGAGTGGATTGCG, generated a 543-bp fragment. The PCR reaction in a GeneAmp PCR system 9700 (Applied Biosystems) used 35 cycles at 94°C 45 s, 66°C 40 s, 72°C 1 min. PCR products (15 µl) were electrophoresed on a 1.8% agarose gel, stained with ethidium bromide and visualized by UV illumination.

WT and PKR^−/−^ mice were infected with early log phase cultures of Mtb H37Rv by aerosol using a Glas-Col Inhalation Exposure System (Glas-Col Inc.). At indicated times, mice were euthanatized by CO_2_ inhalation. The initial inoculum was enumerated by collecting lungs from members of the cohort 24 hr post-infection. Serum was collected for RNI determination and lungs were homogenized in PBS. A portion of the homogenate was reserved for ELISAs. The remainder was serially diluted and plated on Middlebrook 7H11 agar plates (BD Biosciences). CFU were counted after 21days.

For in vitro experiments, bone marrow cells were flushed from femurs and differentiated for 6 days in 150×15 mm petri dishes in DMEM containing 0.29 g/L L-glutamine, 1 mM sodium pyruvate, 10% FBS and 20% L929 fibroblast-conditioned medium. Macrophages were resuspended by incubation in 1 mM EDTA in PBS for 10 min on ice, washed twice with PBS and plated into 24- or 48-well tissue culture plates (Corning). Purified recombinant mouse IFN-gamma, TNF-alpha and IL10 were from R&D Systems. The PKR inhibitor *N*-(2(1*H*-indol-3-yl)ethyl)-4-(2-methyl-*1H*-indol-3-yl)pyrimin-2-amine [33] was initially purchased from Villapharma and subsequently synthesized in the Milstein Chemistry Core Facility at Weill Cornell Medical College.

### Assays for Cytokines and RNI

Macrophages (5×10^5^ in 1 mL of complete DMEM containing 10% L-cell-conditioned medium) were seeded into 24-well tissue culture plates, treated with or without IFN-gamma (10 ng/mL) and infected or not with Mtb at the indicated MOIs. Supernatants were tested for IL10 and TNF-alpha using a Duoset ELISA (R&D Systems). Nitrite was detected by mixing an aliquot of supernatants with an equal volume of Griess's reagent (1% sulfanilamide, 0.1% naphthylethylenediamine dihydrochloride, 2.5% H_3_PO_4_). Absorbance at 550 nm was measured with sodium nitrite as standard. Nitrite content of cell-free medium was subtracted. For assays on serum from Mtb-infected mice, nitrite and nitrate were measured by the Griess reaction after nitrate was reduced to nitrite as described [30].

### Western Blot

Macrophages (5×10^6^) were seeded in 6-well tissue culture plates, pre-treated or not overnight with IFN-gamma (10 ng/mL) and infected or not with Mtb at stated MOIs. At the indicated time, macrophages were lysed and boiled in reducing SDS-PAGE sample buffer containing 100 mM Tris, pH 6.8, 4% SDS, 5% beta-mercaptoethanol, 20% glycerol and 0.2% bromophenol blue. Lysates was subjected to 4–15% gradient gel electrophoresis (Bio-Rad) and transferred to a 0.2 micron pore nitrocellulose membrane (Schleicher & Schuell) in 20% methanol, 25 mM Tris and 192 mM glycine, pH 8.3 and 0.1% SDS. Anti-PKR Ab (sc-1702) for western blot was from Santa Cruz Biotech. Anti-iNOS Ab was from Abcam. Detection was by the Odyssey system (LI-COR Biosciences).

### Real-Time Quantitative RT-PCR

Qiagen furnished RNA extraction kits. Invitrogen synthesized oligonucleotide primers and Biosearch Technologies provided probes labeled with fluorescent amidites (FAM) at the 5′ end and Black Hole Quencher (BHQ) at the 3′ end. Primers and probe sequences for mature iNOS were TGCTCCCTTCCGAAGTTTCTG (forward), TCATGCGGCCTCCTTTGAG (reverse), and AGCAGCGGCTCCATGACTCCCAG (probe). Primers and probe sequences for mature IL10 were AGACCCTCAGGATGCGGC (forward), CCACTGCCTTGCTCTTATTTTCA (reverse), and AGGCGCTGTCATCGATTTCTCCCCT (probe). Primers and probe sequences for mature TNF-alpha were GGCCTCCCTCTCATCAGTTCT (forward), GTGGGCTACAGGCTTGTCA (reverse), and TGGCCCAGACCCTCACACTCAG (probe). Primers and probe sequences for GAPDH were GGGCATCTTGGGCTACACT (forward), GGCATCGAAGGTGGAAGAGT (reverse), and AGGACCAGGTTGTCTCCTGCGA (probe). Primers and probe sequences for nascent iNOS were CTCACGCTTGGGTCTTGTTCA, (forward), CCAAGCAGGAAGACACTCCTAAG (reverse), and AGTAGCCTAGTCAACTGCAAGGTGAGTC (probe). Primers and probe sequences for nascent IL10 were CAGAGCCACATGCTCCTAGAG (forward), TCAGGTGATGGCAGGAAGAAAG (reverse), and CGGACTGCCTTCAGCCAGGT (probe). The primers and probe sequences for nascent TNF-alpha were CACCACGCTCTTCTGTCTACTG (forward), CTGTCCTTCTTGCCCTCCTAAC (reverse), and TGATCGGTCCCCAAAGGGATGA (probe). 100–300 ng RNA was transcribed into cDNA with oligo d(T) in 20 microL using MulLV reverse transcriptase (Applied Biosystems). cDNA was diluted to 200 microL. We performed PCR in 15 microL in an ABI PRISM 7900HT sequence detection system and normalized results to GAPDH.

### mRNA Stability

Primary macrophages were seeded in 24-well tissue culture plates overnight, then stimulated with IFN-gamma (10 ng/mL). Actinomycin D (Sigma-Aldrich) was added 3 h later. Total RNA was extracted at indicated times and transcribed into cDNA with oligo d(T) using MulLV reverse transcriptase (Applied Biosystems). cDNA was amplified by PCR using Taq DNA polymerase (New England BioLabs) and PCR products were analyzed by agarose gel electrophoresis. Primers for iNOS were AGTATAAGGCAAGCACCTTGG (forward) and GCTCTGGATGAGCCTATATTGC (reverse). Primers for IL10 were GAAGACAATAACTGCACCCACT (forward) and TTCATGGCCTTGTAGACACCT (reverse). Primers for TNFα were GACGTGGAACTGGCAGAAG (forward) and CAGCCTTGTCCCTTGAAGAG (reverse). The primers for GAPDH were TGGAGATTGTTGCCATCAACG (forward) and AAGTTGTCATGGATGACCTTGG (reverse). Results were normalized to GAPDH.

### Immunocytology

Primary macrophages were mounted on nitric acid-treated coverslips, pretreated or not with IFN-gamma (10 ng/mL) overnight and then infected or not with Mtb for 24 h. Macrophages were fixed for 2 h with 4% (vol/vol) paraformaldehyde in PBS and permeabilized for 30 min at room temperature with 0.2% saponin in PBS containing 10% (vol/vol) donkey serum. Coverslips were incubated for 30 min at room temperature with anti-PKR antibody (dilution, 1∶50). Cells were rinsed twice with PBS containing 0.2% saponin and stained for 1 h at room temperature with secondary antibody conjugated with Alexa fluor™ 488 (Molecular Probes) (dilution, 1∶500). Coverslips were washed twice with PBS containing 0.2% saponin, once in ddH_2_O and mounted on slides. Images were acquired with an inverted LSM 510 Laser scanning confocal microscope [57] at the Rockfeller University Bio-Imaging Resource Center.

### Apoptosis Assays

Apoptosis of macrophages in vitro was evaluated by TdT-mediated dUTP nick-end labeling (TUNEL) using TMR red *in-situ* cell death detection and by Cell Death Detection ELISA^PLUS^ (Roche Applied Science). For TUNEL, 5×10^4^ primary macrophages per well were seeded into 48-well plates and treated or not with 10 ng/mL of IFN-gamma overnight. Cells were infected with Mtb at MOI 10 for 24 h, fixed with 2% paraformaldehyde in PBS for 2 h, permeabilized with 0.1% Triton x-100 in 0.1% sodium citrate for 2 min on ice and incubated in TdT reaction mixture containing TMR red dUTP for 1 h at 37°C in a dark, humidified chamber. Total cell number was assessed by following incubation with 4′,6-diamidino-2-phenylindole (DAPI) for 5 min. The cells were viewed at room temperature using an Olympus IX-70 inverted fluorescent microscope. The apoptosis ELISA measured cytoplasmic histone-associated DNA fragments. Macrophages (5×10^5^) were incubated with or without IFN-gamma (10 ng/mL) in 24-well plates at 37°C overnight and then infected with Mtb at MOI 5, 10 or 20 for 24 h before assay of cell lysates following the manufacturer's specifications. The relative amount of apoptosis was calculated as a ratio of the difference in absorbance at 405 nm and 490 nm of infected macrophages to that of uninfected macrophages.

Apoptotic macrophages in lungs of Mtb-infected mice were identified as described [58]. Formalin-fixed, paraffin-embedded lung sections were deparaffinized, rehydrated, treated with 0.1% Triton X100 in PBS for 15 min at room temperature, washed with PBS, blocked with 1.5% donkey serum in PBS for 1 h at 37°C in a humidified chamber, incubated with 1∶500 anti-macrophage antibody (AIA, Accurate Chemical & Scientific) overnight at 4°C and with 1∶500 secondary antibody conjugated with Alexa fluor™ 488 (Molecular Probes) for 1 h at room temperature before TUNEL staining followed by DAPI staining. Fluorescent images were captured by an Olympus IX-70 inverted fluorescent microscope.

### PKR Autophosphorylation

The protocol used in [44] was the kind gift of Dr. Li-Chung Hsu, National Taiwan University. Lysis buffer contained 50 mM Tris-HCl pH 7.5, 250 mM NaCl, 3 mM EDTA, 3 mM EGTA, 1% Triton X-100, 0.5% NP-40, 10% glycerol, 10 mM NaF and was supplemented before use with 2 mM DTT, 1 mM PMSF, 2 mM pNPP, 20 mM beta-glycero-3-phosphate,1 mM Na_3_VO_4_ and protease inhibitor cocktail (Roche). The kinase buffer was prepared at 10× with 200 mM Tris-HCl pH 7.5, 500 mM KCl, 20 mM MgCl_2_, 20 mM MnCl_2_, and 50% glycerol. The reaction buffer was 1× kinase buffer with 20 mM beta-glycero-3-phosphate, 10 mM pNPP, 20 mM ATP, 1 mM DTT. Macrophages (1×10^7^) treated or not with 10 ng/mL IFN-gamma were washed three times in PBS and lysed in 1 mL of lysis buffer for 5–10 min at 4°C. Debris was removed by centrifugation at 13,000 g for 10 min at 4°C and supernatant aliquotted, its protein concentration determined by the BCA method (BioRad) and stored at −80°C. For assay, 200 micrograms of protein per sample was transferred to a fresh tube and brought to 400 microL in lysis buffer. Anti-PKR Ab (10 microL) (Santa Cruz, M-515) was added and the samples each rotated overnight with 15 mL of a 50% slurry of protein A beads at 4°C. Immune complexes were collected by centrifugation for 1 min at 13,000 g, washed twice in lysis buffer and once with kinase buffer and resuspended in 25 µl reaction buffer containing 10 microCi [gamma-P^32^]-ATP. After 30 min at 30°C, reactions were stopped with 100 microL of lysis buffer. The beads were collected by centrifugation for 1 min, mixed with 15 microL of 2× SDS sample buffer and heated at 100°C for 5 min. The supernatants were analyzed by 4–15% SDS-PAGE (Bio-Rad). When the dye front ran off the gel, the bottom of the gel containing the free isotope was removed and the gel dried for placement in a Phosphorimager.

## Supporting Information

Figure S1 **Lack of effect of PKR on activation of ERK1/2, p38, STAT1 and STAT3 by IFN-gamma in primary macrophages.** Primary macrophages from wild type and PKR−/− mice were cultured at 37°C overnight and treated with IFN-gamma (10 ng/mL) for 24 h. Cell lysates were separated by SDS-PAGE and analyzed by western blotting using antibodies against phospho-ERK1/2, phospho-p38, phospho-STAT1 and phospho-STAT3. Beta-tubulin was used as a loading control.
(PDF)

Figure S2 **PKR facilitates IFN-gamma-induced binding of Stat1 to iNOS GAS.** EMSA was performed as described in Methods. Infrared Dye 700-labeled 15-base pair oligonucleotide containing the iNOS GAS and 5 micrograms of nuclear extract were used in each lane. (A) 2×107 primary macrophages from wild type and PKR−/− mice were treated with IFN-gamma (10 ng/mL) for the indicated time. −, no addition of nuclear extract. Solid arrowhead indicates Stat1-specific binding. (B) 2×107 primary macrophages from wild type and PKR−/− mice were treated with IFN-gamma (10 ng/mL) for 15 min. −, no addition of nuclear extract. +, only addition of nuclear extract. For other lanes, the nuclear extract was pre-incubated with antibody against Stat1, an excess of unlabeled iNOS GAS and antibody against Stat3, respectively. Supershifted band is indicated by solid arrowhead.
(PDF)

Figure S3 **Confirmation of PKR deficiency in macrophages from knock-out mice.** Primary macrophages were from wild type (WT) C57BL/6 mice or PKR−/− mice derived from founders kindly provided by C. Weissmann (Yang et al.). (A) Immunoblot for PKR with beta-tubulin as a loading control. (B) Autophosphorylation of PKR at indicated times after exposure to poly-IC (10 micrograms/mL).
(PDF)
