# Correction: Improved Control of Tuberculosis and Activation of Macrophages in Mice Lacking Protein Kinase R

**DOI:** 10.1371/journal.pone.0205424

**Published:** 2018-10-05

**Authors:** Kangyun Wu, Jovanka Koo, Xiuju Jiang, Ran Chen, Stanley N. Cohen, Carl Nathan

Following publication, the authors conducted additional experiments to determine the genetic background of PKR regulatory domain mutant mice used in this study [1], and in a related article demonstrated that some phenotypes were not replicated when using a different PKR mutant line on a different genetic background [2].

• The genetic background of the PKR regulatory domain mutant mice was not known to the authors at the time the in vivo experiments were conducted for the *PLOS ONE* article, and so was not reported [1]. Following the publication of this work, the authors conducted SNP genotyping analyses and determined that PKR regulatory domain mutant mice used in the experiments were on a 98.9% 129S1/SvImJ genetic background. Hence, for some of the experiments reported in the *PLOS ONE* article, the genetic background is different between mutant and control animals (Figures 1–5).

• Following publication of the *PLOS ONE* article [1], the authors repeated experiments *in vitro* using bone-marrow derived macrophages and in vivo in a mouse model of tuberculosis infection using a different PKR-deficient mouse line harboring a catalytic domain mutation [3] instead of a regulatory domain mutation [4]. In these follow-up experiments, both mutant and control animals were on a C57Bl/6J background. These experiments yielded different results than those reported in the *PLOS ONE* article: the authors did not observe a statistically significant difference in CFU in lung between WT and PKR-deficient mice in the mouse model of Mtb infection, as reported in detail in [2]. While these findings did not support the conclusions of the *PLOS ONE* article, they were not based on exact experimental replications, as noted in the following table.

**Table:** 

	*PLOS ONE* experiment	*EJI* experiment
Origin of PKR mutant lines	Regulatory domain knockout as generated by Yang YL *et al*. *EMBO J*. *1995* [4]	Catalytic domain knockout as generated by Abraham N *et al*. *J*. *Biol Chem*. *1999* [3]
Genetic background	Wild-type (WT) control: C57BL/6J Protein kinase R (PKR)–deficient: 98.9% 129S1/SvImJ	WT control: C57BL/6J PKR-deficient: C57BL/6J
Infection protocol (The Glas-col Inhalation Exposure instrument was different in the two experiments as the two experiments were conducted in different BSL3 facilities.)	Mtb (100–200 CFU) was administered for the aerosol infection on day 0. On day 1 a mean of 30 CFU were recovered from lungs of WT mice and 37 CFU from lungs of PKR-deficient mice.	Mtb (100–200 CFU) was administered for the aerosol infection on day 0. On day 1 a mean of 127 CFU were recovered from lungs of WT mice and 137 CFU from lungs of PKR-deficient mice.
Th timing of outcome assessments (Day 14–21 and day 57–168 can be defined as acute phase and chronic phase of infection respectively. Therefore, the stages assayed were same in *PLOS ONE* and *EJI* experiments).	Post-infection days 21, 70 and 168. *The CFU burden in lungs of PKR-deficient mice was 4- to 10-fold lower than in WT mice in the chronic phase of infection.	Post-infection days 14, 29, 57 and 120. *The CFU burden in lungs of PKR-deficient mice was not distinguishable from that of WT mice in the chronic phase of infection.

• The differences between the *PLOS ONE* and *EJI* experiments are tabulated above. The macrophage experiments in *PLOS ONE* article [1] used WT mice on a C57BL/6J background and regulatory domain PKR KO mice on a 129S1/SvImJ background. The macrophage experiments in the *EJI* article [2] used WT mice on a C57BL/6J background and both regulatory domain and catalytic domain PKR KO mice, each on a C57BL/6J background, and observed that PKR sufficiency or deficiency did not impact macrophage activation. The *EJI* article also compared WT mice on a 129S1/SvImJ background and regulatory domain PKR KO mice on a 129S1/SvImJ background, similar to the mice used in [1], and observed that interferon-gamma alone caused macrophage activation to similar levels in both WT and mutant, suggesting that heightened macrophage activation could be a background effect. In the *EJI* article it was observed that there was no statistically significant difference in Mtb CFU burden between WT and catalytic domain PKR KO mice, both on the C57BL/6J background. The *EJI* study casts doubt on the candidacy of PKR as a target for HDT of TB.
