# Supplementary material for: Improved Control of Tuberculosis and Activation of Macrophages in Mice Lacking Protein Kinase R
Source: PLoS One. 2012 Feb 16;7(2):e30512. doi: 10.1371/journal.pone.0030512 (PMC3281035; doi:10.1371/journal.pone.0030512)
Supplement: Figure S1 — Lack of effect of PKR on activation of ERK1/2, p38, STAT1 and STAT3 by IFN-gamma in primary macrophages. Primary macrophages from wild type and PKR−/− mice were cultured at 37°C overnight and treated with IFN-gamma (10 ng/mL) for 24 h. Cell lysates were separated by SDS-PAGE and analyzed by western blotting using antibodies against phospho-ERK1/2, phospho-p38, phospho-STAT1 and phospho-STAT3. Beta-tubulin was used as a loading control. (PDF) [file pone.0030512.s001.pdf]

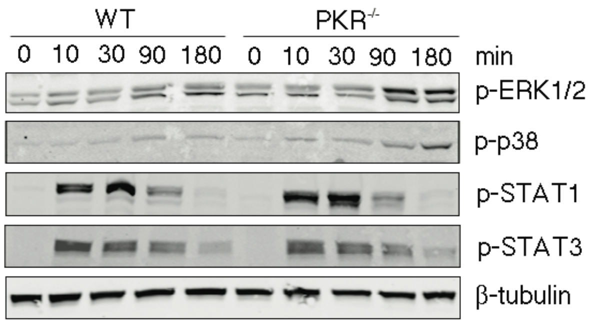

Figure S1. Lack of effect of PKR on activation of ERK1/2, p38, STAT1 and STAT3 by IFNg in primary macrophages.

Primary macrophages from wild type and $PKR^{-/-}$ mice were cultured at 37˚C overnight and treated with IFNg (10 ng/mL) for 24 h. Cell lysates were separated by SDS-PAGE and analyzed by western blotting using antibodies against phospho-ERK1/2, phospho-p38, phospho-STAT1 and phospho-STAT3. ß-tubulin was used as a loading control.
