# Supplementary material for: Improved Control of Tuberculosis and Activation of Macrophages in Mice Lacking Protein Kinase R
Source: PLoS One. 2012 Feb 16;7(2):e30512. doi: 10.1371/journal.pone.0030512 (PMC3281035; doi:10.1371/journal.pone.0030512)
Supplement: Figure S2 — PKR facilitates IFN-gamma-induced binding of Stat1 to iNOS GAS. EMSA was performed as described in Methods. Infrared Dye 700-labeled 15-base pair oligonucleotide containing the iNOS GAS and 5 micrograms of nuclear extract were used in each lane. (A) 2×10^7^ primary macrophages from wild type and PKR−/− mice were treated with IFN-gamma (10 ng/mL) for the indicated time. −, no addition of nuclear extract. Solid arrowhead indicates Stat1-specific binding. (B) 2×10^7^ primary macrophages from wild type and PKR−/− mice were treated with IFN-gamma (10 ng/mL) for 15 min. −, no addition of nuclear extract. +, only addition of nuclear extract. For other lanes, the nuclear extract was pre-incubated with antibody against Stat1, an excess of unlabeled iNOS GAS and antibody against Stat3, respectively. Supershifted band is indicated by solid arrowhead. (PDF) [file pone.0030512.s002.pdf]

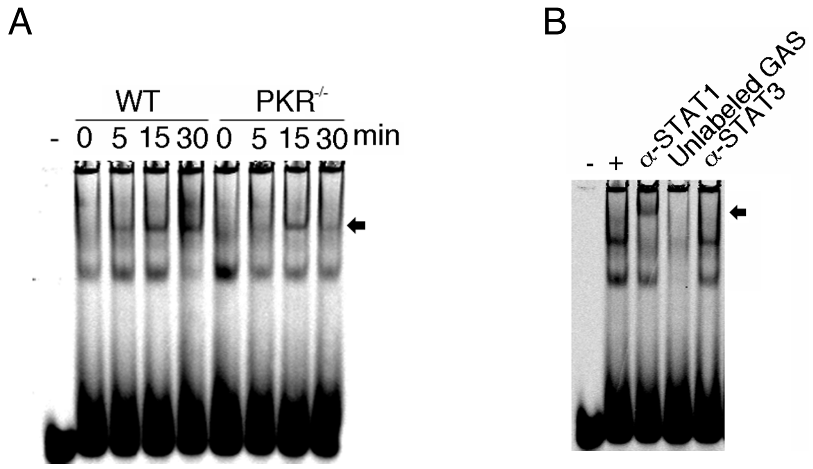

Figure S2. PKR facilitates IFNg-induced binding of Stat1 to iNOS GAS.

EMSA was performed as described in Methods. Infrared Dye 700-labeled 15-base pair oligonucleotide containing the iNOS GAS and 5 µg of nuclear extract were used in each lane.

(A) 2 x 10^7 primary macrophages from wild type and $PKR^{-/-}$ mice were treated with IFNg (10 ng/mL) for the indicated time. -, no addition of nuclear extract. Solid arrowhead indicates Stat1-specific binding.

(B) 2 x 10^7 primary macrophages from wild type and $PKR^{-/-}$ mice were treated with IFNg (10 ng/mL) for 15 min. -, no addition of nuclear extract. +, only addition of nuclear extract. For other lanes, the nuclear extract was pre-incubated with antibody against Stat1, an excess of unlabeled iNOS GAS and antibody against Stat3, respectively. Supershifted band is indicated by solid arrowhead.
