# Supplementary material for: Improved Control of Tuberculosis and Activation of Macrophages in Mice Lacking Protein Kinase R
Source: PLoS One. 2012 Feb 16;7(2):e30512. doi: 10.1371/journal.pone.0030512 (PMC3281035; doi:10.1371/journal.pone.0030512)
Supplement: Figure S3 — Confirmation of PKR deficiency in macrophages from knock-out mice. Primary macrophages were from wild type (WT) C57BL/6 mice or PKR−/− mice derived from founders kindly provided by C. Weissmann (Yang et al.). (A) Immunoblot for PKR with beta-tubulin as a loading control. (B) Autophosphorylation of PKR at indicated times after exposure to poly-IC (10 micrograms/mL). (PDF) [file pone.0030512.s003.pdf]

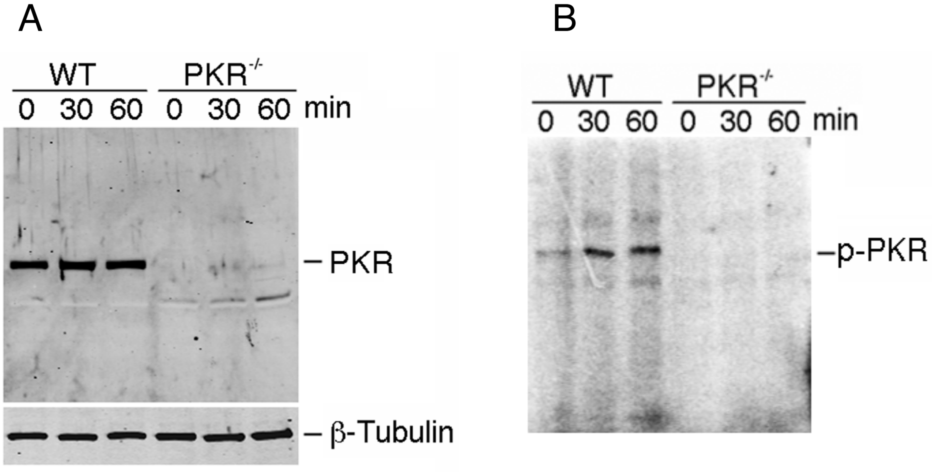

Figure S3. Confirmation of PKR deficiency in macrophages from knock-out mice.

Primary macrophages were from wild type (WT) C57BL/6 mice or $PKR^{-/-}$ mice derived from founders kindly provided by C. Weissmann (Yang et al.).

(A) Immunoblot for PKR with b-tubulin as a loading control.

(B) Autophosphorylation of PKR at indicated times after exposure to poly-IC (10 µg/mL).
